# Larval Cytotoxic and Subacute Toxicity of *Gardenia ternifolia*, *Rourea coccinea*, and *Cassytha filiformis* Used in Traditional Medicine of Benin (West Africa)

**DOI:** 10.1155/2020/8843575

**Published:** 2020-10-28

**Authors:** E. Agbodjento, J. R. Klotoé, T. I. Sacramento, T. V. Dougnon, E. Déguenon, J. Agbankpé, K. Fabiyi, P. Assogba, M.-P. Hounkanrin, R. Akotegnon, T. J. Dougnon, J.-M. Atègbo

**Affiliations:** ^1^Research Unit in Applied Microbiology and Pharmacology of natural substances, Research Laboratory in Applied Biology, Polytechnic School of Abomey-Calavi, University of Abomey-Calavi, 01P.O.Box 2009, Cotonou, Benin; ^2^Normal High School of Natitingou, National University of Sciences, Technology, Engineering and Mathematics, P.O. Box 72, Natitingou, Benin; ^3^School of Management and Operation of Livestock Systems, National University of Agriculture, Ketou, Benin; ^4^Laboratory of Molecular Physiopathology and Toxicology, Faculty of Sciences and Techniques, University of Abomey-Calavi, 01 P.O.Box 526, Cotonou, Benin

## Abstract

The use of medicinal plants in traditional medicine is a common practice in developing countries. However, this unregulated or irrational use may pose a risk of toxicity to humans in the short and/or long term. Recent studies reported interesting ethnopharmacological, antioxidant, and phytochemical data on some medicinal plants used in the traditional treatment of male infertility in Benin. Unfortunately, very little data exist on the long-repeated dose toxicity of these medicinal plants. This study was aimed at evaluating the larval cytotoxicity and subacute toxicity of the hydroethanolic extract of *Cassytha filiformis* whole plant, *Gardenia ternifolia* roots, and *Rourea coccinea* leaves. The subacute toxicity of these plants was evaluated in male Wistar albino rats at three different doses (200, 400, and 800 mg/kg) according to the OECD 407 guidelines. Hematological and biochemical examinations and the histological study of the liver and kidneys were carried out. Larval cytotoxicity was assessed by the sensitivity of *Artemia salina* larvae to different concentrations of the studied plants extracts. The mean lethal concentration (LC_50_) was determined by the probit method. Subacute toxicity data indicated that there was no mortality or structural alterations of the liver and kidneys in the lot of treated animals. However, significant alterations in certain hematological and biochemical parameters (hematocrit, ASAT, and uremia) were noted. These abnormalities were observed in the lot of rats treated with *Rourea coccinea* and *Cassytha filiformis* extracts. Larval cytotoxicity data indicate that the studied plants extracts are not cytotoxic (LC_50_ > 0.1 mg/mL). These data suggest that the use in traditional medicine of studied plants at high doses and repeated over a long period of time requires special attention.

## 1. Introduction

Traditional medicine has an important place in primary healthcare for people throughout the world. According to the WHO, more than 80% of the African and Western population use this medicine for a multitude of reasons and for their well-being [1]. This medical practice based on the use of medicinal plants is an ancestral tradition handed down from generation to generation [2, 3]. The population uses remedies without guaranteeing the safety of the medicinal plants that compose them. In the literature, reports have mentioned that poorly matched combinations of plants present toxicity risks [4, 5]. These data suggest that the toxicological characterization of medicinal plants is of paramount importance for safe use in phytotherapy. Reports in the literature have demonstrated the short-, medium-, and long-term toxic effects of medicinal plants [6, 7].

Africa is known for the richness of its flora, which is conducive to scientific studies for a better valuation of these natural resources. Benin has an interesting ethnobotanical potential. Akoègninou et al. [8] estimated the Beninese flora at more than 2807 plant species. *Gardenia ternifolia* (*G. ternifolia*), *Cassytha filiformis* (*C. filiformis*), and *Rourea coccinea* (*R. coccinea*) are three plants of Beninese flora used in the traditional treatment of male infertility [9]. Recent studies reported the antioxidant potential and the richness of these three plants in polyphenols and flavonoids, which often are responsible for their medicinal properties [10]. In the African and Western pharmacopoeias, *G. ternifolia*, *C. filiformis*, and *R. coccinea* have numerous ethnopharmacological applications.

In Togo, *G. ternifolia* leaves are traditionally used in the treatment of hypertension [11, 12] and diabetes [13]. In RD Congo, the same leaves of the plant are indicated in the management of sickle cell disease [14], cancer [15], and hemorrhoids [16]. The roots and fruits of *G. ternifolia* are used, respectively, in Ethiopia [17] and Sudan [18] in the treatment of malaria. In the Guinean pharmacopoeia, all parts of *G. ternifolia* are known to have powerful antibiotic, hypotensive, and antidiabetic properties [19, 20]. In Nigeria, *G. ternifolia* leaves are used in the treatment of liver necrosis [21]. In Senegal, it has been reported that the leaves of the plant are used to treat diarrhea [22], while its roots are used to treat tooth decay, hemorrhoids, leprosy, and rheumatism [23]. These therapeutic virtues of *G. ternifolia* are attributed to alkaloids, anthocyanins, flavonoids, phenols, saponins, tannins, steroids, and terpenoids identified in different parts of the plant [17, 24].

*R. coccinea* is a medicinal plant whose therapeutic virtues are widely recognized in West Africa. In Nigeria, the leaves are used in the treatment of diabetes [25], fever [26], diarrhea [27], and sexually transmitted infections [28]. In Benin, the plant is indicated in the treatment of malaria, male and female infertility, and sexual asthenia [8, 29]. In Togo, the different medicinal uses of the leaves concern dysmenorrhea, hypertension, primary and secondary sterility, tachycardia, rheumatism, hemorrhages, gonorrhea, and mucus [30, 31]. The roots of *R. coccinea* are used in the treatment of anemia, sexual asthenia, and male and female sterility [30]. In Côte d'Ivoire, all parts of the whole plant are used in the treatment of sore throats and muscular and rheumatic pain [32]. These therapeutic uses of *Rourea coccinea* could be explained by the effect of saponins, tannins, steroids, reducing sugar, glycosides, flavonoids, anthraquinone, and alkaloids identified as secondary metabolites in different parts of the plant [28, 33, 34].

As for *C. filiformis*, its therapeutic virtues are recognized in several pharmacopoeias throughout the world. In Polynesia, several people use *C. filiformis* in the treatment of cancers [35]. In northern Nigeria, *C. filiformis* is used in the treatment of diabetes mellitus [36]. In Benin, the plant is indicated in the treatment of gastric ulcers, haemorrhoids, and cough [37]. Secondary metabolites such as flavonoids, phenols, alkaloids, tannins, steroids, and saponins identified in different raw extracts of *Cassytha filiformis* would be responsible for these medicinal properties of the plant [38, 39].

The above evidence attests to the importance of *G. ternifolia*, *C. filiformis,* and *R. coccinea* in traditional African and Western medicine. Unfortunately, despite this frequency of use of these plants for health purposes, it must be noted that rare or nonexistent toxicity tests have explored their safety following long repeated use at different doses. Literature data indicate that the toxicity of medicinal plants can also be established in long-repeated small doses [40]. In addition, in traditional medicine, the management of pathologies most often requires the use of medicinal potions over a long period of time.

This study was initiated to fill this gap in the scarcity of scientific data on the repeated dose toxicity of selected plants. It aimed to evaluate larval cytotoxicity and subacute toxicity of *G. ternifolia* roots, *C. filiformis* whole plant, and *R. coccinea* leaves in rats to predict its safety in human use. Rats are recommended lower level of animals for toxicity studies to extrapolate to human biology according to the Organization for Economic Cooperation and Development (OECD) safety study guidelines [41, 42]. The finding of the study could also help to guide optimization and validation of the traditional use of these medicinal plants.

## 2. Materials and Methods

### 2.1. Study Material

#### 2.1.1. Plant Material

Plant materials used were constituted of *G. ternifolia* roots, *C. filiformis* whole plant, and *R. coccinea* leaves. These plant organs were collected in March 2019 in the municipality of Za-Kpota and Djidja (Zou department, southern Benin) and certified at the Benin National Herbarium under the voucher number YH261/HNB for *Rourea coccinea*, YH262/HNB for *Cassytha filiformis*, and YH263/HNB for *Gardenia ternifolia*. The harvest time of these plants studied was chosen based on the foliage time of each plant. In addition, it is during these periods that the traditional healers collect these plant samples for use in Beninese pharmacopeia.

#### 2.1.2. Animal Material

The animal materials were constituted by *Artemia salina* eggs (ARTEMIO JBL D-67141 Gmbh Neuhofem) and male Wistar rats. *Artemia salina*' eggs (ARTEMIO JBL D-67141 Gmbh Neuhofem), acquired from the Applied Hydrobiology's Laboratory of Agricultural Sciences' Faculty at University of Abomey-Calavi (UAC, Benin), were used for the larval cytotoxicity test. Male Wistar albino rats aged at least three months with a body weight between 150 g and 200 g were used as the animal material for the subacute toxicity test. These animals have been acquired at the animal farm of the Institute of Applied Biomedical Sciences of the University of Abomey-Calavi of Benin. They have been housed in cages covered with wood chips and acclimatized for 2 weeks before experiment at the animal farm of the Research Unit in Applied Microbiology and Pharmacology of natural substances (URMAPha). The rats had free access to water and food. Animal Research Review Panel and Animal Welfare Unit regulations of temperature and lighting systems were maintained with a room temperature of 20–26°C and regular light cycles of 12 hours light/dark. All methods and protocols used in this study were observed following established public health guidelines “Guide for Care and Use of Laboratory Animals.”

### 2.2. Ethical Approval

This study is part of a thesis. The committee of the “Doctoral School Life and Earth Science (ED-SVT)” of the University of Abomey-Calavi (UAC-Benin) under the number 10185509 has authorized this study.

### 2.3. Methods of Study

#### 2.3.1. Production of Plant Extracts

*R. coccinea* leaves, *C. filiformis* whole plant, and *G. ternifolia* roots collected were washed thoroughly with tap water to remove any form of dirt and dried in the shade at room temperature at the Research Unit in Applied Microbiology and Pharmacology of natural substances (URMAPha). They were then powdered with an electric mill. From this powder, a hydroethanolic extraction was carried out according to the methodology described by Klotoé et al. [10]. Fifty (50) grams of powder were macerated in 500 mL of the mixed solvent with equal volume of distilled water and 96% ethanol. The mixture was continuously stirred for 72 hours at room temperature. The homogenate obtained has been filtered three times on hydrophilic cotton and once on Wittman paper No. 1. The filtrate obtained has been evaporated at the temperature of 40°C in an oven until a dry mass that represents the extract. The extract obtained has been weighed and used to evaluate the extraction yield (EY) and then kept in the refrigerator at 4°C. The following formula was used:

(1) $$\begin{array}{c} \text{EY}=\frac{\text{Mass of the extract after evaporation of the solvent}}{\text{Mass of the powder of the plant species used for extraction}}\times 100\text{.} \end{array}$$

#### 2.3.2. Larval Cytotoxicity Test

The cytotoxic effect of the studied plants hydroethanolic extract was evaluated on brine shrimp larvae. This is a preliminary nonclinical toxicity test performed according to the method described by Dougnon et al. [43]. *Artemia salina* larvae were obtained by hatching 10 mg of *Artemia salina* eggs under continuous agitation in 1 L of seawater for 48 h. Order stock extract solution's 2 dilution series with a concentration of 20 mg/mL were carried out in order to have an increasing scale concentration. 1 mL of each diluted solutions was added to 1 mL of sea water containing 16 live larvae. A control solution without the extract was prepared under same conditions. All solutions were incubated under agitation for 24 hours. Counting dead larvae number in each solution under an optical microscope produced a representative curve of the number of surviving larvae versus the concentration of the extract. The data (concentration-response) were log-transformed, and the LC_50_ (mean lethal concentrations) was determined. To assess the larval toxicity of the extract, the correlation grid associating the degree of toxicity with LC_50_ proposed by Mousseux was used [44]. According to this grid, if the LC_50_ value is greater than 0.1 mg/mL, the extract is declared nontoxic. If this value is between 0.1 and 0.5 mg/mL, the extract is weakly toxic, and if the LC_50_ is less than 0.5 mg/mL, the extract is toxic.

#### 2.3.3. Subacute Toxicity Test

This study was conducted according to the OECD 407 guidelines [41], a method by subacute toxicity class. Forty (40) male Wistar albino rats divided by weight into ten (10) lots (9 test lots and one control lot) of four rats each were used in this study. The animals in the test lots were treated at the same time for a period of 28 days by esophageal gavage using three gradual doses (200, 400, and 800 mg/kg) selected from the active pharmacological dose. Previous scientific investigations on these medicinal plants agree on 200 mg/kg as their pharmacologically active dose [31, 45, 46]. The control lot is treated with distilled water under same conditions. Animals in each lot were individually marked with permanent markers.

  Lot 1: distilled water, 1 mL/100 g (control)
  Lot 2: *G. ternifolia,* 200 mg/kg
  Lot 3: *G. ternifolia,* 400 mg/kg
  Lot 4: *G. ternifolia,* 800 mg/kg
  Lot 5: *C. filiformis,* 200 mg/kg
  Lot 6: *C. filiformis,* 400 mg/kg
  Lot 7: *C. filiformis,* 800 mg/kg
  Lot 8: *R. coccinea,* 200 mg/kg
  Lot 9: *R. coccinea,* 400 mg/kg
  Lot 10: *R. coccinea,* 800 mg/kg

During the experiment, the animals were weighed every seven days (day 0, day 7, day 14, day 21, and day 28), followed, and observed individually twice a day (morning and evening). A data collection sheet was prepared for each rat to collect possible signs of toxicity (changes in skin, hair, edema, walking back, respiratory difficulties, morbidity, and mortality). At the end of treatment, rats were deprived of food the last night before sampling. Blood samples (day 0 and day 28) were performed by puncture at the retroorbital sinus for all animals under ether anesthesia. The blood sample is recovered from two types of tubes, one containing EDTA and one dry without anticoagulant. The EDTA tube sample is intended for hematological analysis. Dry tubes were centrifuged at 4000 rpm for 10 minutes, and the serum obtained is kept at −20°C for biochemical parameter analysis. After sampling, two animals in each lot were sacrificed under anesthesia with ether for the collection of organs such as the liver and kidney. These organs were rinsed with a 0.9% saline water and fixed in 10% buffered formaldehyde.

*(1) Hematological Examinations*. These examinations were performed using the SYSMEX KX 21N automaton using the method used by Sodipo et al. [47]. These examinations included red and white blood cell counts, hemoglobin level, hematocrit, mean globular volume (MGV), mean corpuscular hemoglobin content (MCH), and determination of mean corpuscular hemoglobin concentration (MCHC).

*(2) Biochemical Examinations*. The biochemical tests were performed at the Research Unit in Applied Microbiology and Pharmacology of natural substances (URMAPha). This consisted to the determination of urea, creatinine, aspartate aminotransferase (ASAT), and alanine aminotransferase (ALAT).

*(3) Histological Examinations*. Histological sections of the liver and kidneys were performed at the Histopathology Laboratory of the Institute of Applied Biomedical Sciences (ISBA) of the University of Abomey-Calavi. The pathomorphological study consisted of hematoxylin-eosin staining of thin sections of 5 *μ*m thicknesses. It is routine staining after which the nuclei, stained by hematoxylin, appear dark blue and the cytoplasm, stained by eosin, appear pink. The microscopic observation of these sections was carried out with the ZEISS camera microscope at different magnifications, so that only the most representative photographs were selected.

#### 2.3.4. Data Analysis

The obtained data were subjected to statistical analysis using the SPSS 26.0 and Graph Pad Prism 7 software. Quantitative variables were presented as mean and standard deviation. Qualitative variables were presented in percentages. The probit analysis was used for LC_50_ determination. The student test was used to compare the values of the different biochemical, hematological, and weight gain parameters of the treated animals with those of the control lot. The significance threshold was set at 5%.

## 3. Results

The data are obtained in this study related to the extraction yield, larval cytotoxicity, and different parameters of subacute toxicity.

### 3.1. Extraction Yield

**Figure 1:**
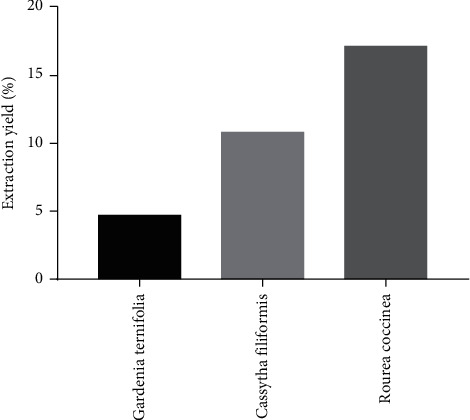
Extraction yield.

The extraction yield obtained for hydroethanolic extraction for the studied plants is shown in Figure 1. From this figure, it emerges that the hydroethanolic extract of *R. coccinea* gave a better yield than the other two plant organs. The lowest yield was obtained with *G. ternifolia* roots.

### 3.2. Larval Cytotoxicity

**Figure 2:**
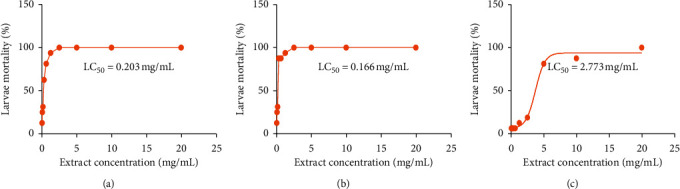
Sensitivity of *Artemia salina* larvae to the studied plants. (a) *Cassytha filiformis*; (b) *Rourea coccinea*; (c) *Gardenia ternifolia*; LC_50_, lethal mean concentration.

The sensitivity of *Artemia salina* larvae to different concentrations of the hydroethanolic extract of the plants studied is presented in Figure 2. The results show an increasing mortality of *Artemia salina* larvae as the concentration of extracts from the plants studied increases. The obtained LC_50_ was 0.203 mg/mL for *C. filiformis*, 0.166 mg/mL for *R. coccinea*, and 2.773 mg/mL for *G. ternifolia*, respectively. These LC_50_ values reported at the scale of Mousseux [44] suggest that at the concentrations tested, the hydroethanolic extract of the plants studied is noncytotoxic (LC_50_ > 0.1 mg/mL).

### 3.3. Subacute Toxicity

#### 3.3.1. Evolution of Animal Weight

**Figure 3:**
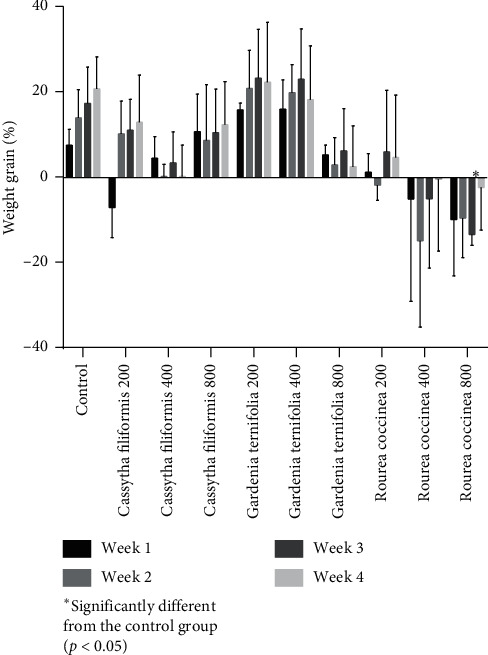
Evolution of animal bodyweight during the experimental period.

The evolution of the weight of the animals during the experimental period is shown in Figure 3. In general, it is noted that the treatments performed have an influence on the weight growth of animals. This influence varies according to the plants studied and the period of exposure of the animals to the different plant extracts. Thus, compared to the weight change control group rats, a nonsignificant weight growth was observed throughout the experimental period for treated rats with the three doses of *G. ternifolia* (*p* > 0.05). The same situation is observed for the treated animals with *C. filiformis*. However, for *R. coccinea*, a variable influence of dose and time (treatment week) on animal weight growth was observed. Thus, at the 200 mg/kg, a nonsignificant weight loss was observed after two weeks of treatment before a resumption of weight growth in the last two weeks. At doses of 400 and 800 mg/kg, a nonsignificant loss of animal bodyweight was observed for treatments performed. This weight loss was significant during the last week of treatment for rats treated at 800 mg/kg dose (*p* < 0.05).

#### 3.3.2. Effect of Plant Extracts on Hematological Parameters of Treated and Control Rats

Table 1 provides information on the effect of the studied plants extracts on the hematological parameters of the animals of the different lots. The analysis of this table shows a significant reduction in the hematocrit level for animals treated with extracts of *C. filiformis* and *R. coccinea* compared to untreated rats for the erythrocyte data (*p* < 0.05). In addition, a nonsignificant dose-dependent reduction in the red blood cell count, mean cell volume, hemoglobin level, and mean corpuscular hemoglobin concentration was also noted for the treated rats with *R. coccinea* (*p* > 0.05). For *C. filiformis*, a nonsignificant reduction in the mean blood volume and mean body hemoglobin content accompanies the significant reduction in the hematocrit. These data suggest microcytic hypochromic anemia in Wistar rats treated with *R. coccinea* and *C. filiformis*. For *G. ternifolia*, no significant effect on hematological parameters is reported for the studied doses.

#### 3.3.3. Effect of the Studied Plants Extracts on Biochemical Parameters

Renal parameters (urea and creatinine) were examined to explore renal function. Alanine aminotransferase (ALAT) and aspartate aminotransferase (ASAT) were considered in the exploration of the liver function (Table 2). Analysis of the data in this table indicates that animals treated with 400 mg/kg and 800 mg/kg of the plants studied showed a significant increase in uremia (*p* < 0.05). However, no significant influence on creatinemia of animals was noted (*p* > 0.05).

In addition, concerning to liver parameters, a significant reduction in serum ASAT was observed in animals treated with *R. coccinea* and *C. filiformis* particularly at doses of 400 mg/kg and 800 mg/kg. However, no significant difference was noted for this parameter in rats treated with *G. ternifolia*. On ALAT, except the animals treated with *Cassytha filiformis* 200 mg/kg and *Gardenia ternifolia* 400 mg/kg, no significant influence was noted for this hepatic parameter of animals in other lots (*p* > 0.05).

#### 3.3.4. Histological Examination of the Liver and Kidney

**Figure 4:**
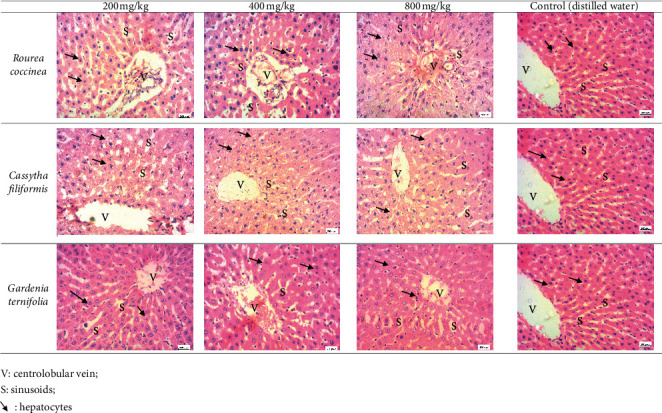
Liver histology of treated and untreated rats (×400 magnification).

**Figure 5:**
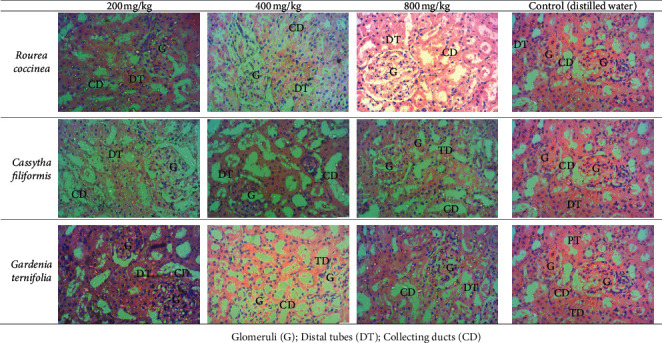
Kidneys histology of treated and untreated rats (×400 magnification).

Figure 4 shows the liver histology of treated and untreated animals. From this figure, it appears that the hepatic parenchyma of treated rats has the same appearance as that of control rats. Hepatocytes (arrows) have a normal appearance and are arranged in cords separated by sinusoids (*s*). These sinusoids are arranged around the centrolobular vein (*V*).  Figure 5 shows the renal histology of the treated animals and the control group. Analysis of the data in this figure indicates that the renal parenchyma of the treated rats has a typical architecture than that of the control group rats. Glomeruli (*G*), distal tubes (DT), and collecting ducts (CD) are well identifiable. So, the extracts did not affect the kidney structures.

## 4. Discussion

Medicinal plants and their derivatives are used as an alternative in healthcare by the majority of people in developing countries. Despite its medicinal uses, very few toxicological studies have been conducted on these medicinal plants. The purpose of this study was to evaluate the toxicological effect of *R. coccinea*, *C. filiformis*, and *G. ternifolia* through larval cytotoxicity and subacute toxicity tests. The cytotoxic effect of the extracts evaluated according to the *Artemia salina* model was used as a preliminary screening to determine the safety of the plants studied. The results indicated that plants studied at tested concentrations are noncytotoxic to the larvae of *Artemia salina*. Lagarto Parra et al. [48] demonstrated a good correlation (*r* = 0.85; *p* < 0.05) between this larval cytotoxicity test and toxicological effects on an entire animal. However, such extrapolation is strongly discussed in the literature [49]. To support this data and to explore the safety of the plants studied following daily exposure to repeated doses in the Wistar albino rat, the subacute toxicity test was conducted. The results of this toxicological screening indicated that prolonged exposure of rats to different doses could affect weight growth, biochemical, and hematological parameters. For weight growth of animals during the period of experimentation, a loss of bodyweight should be reported in rats treated with *R. coccinea* extract at high doses (400 and 800 mg/kg). These data can be explained by the anorexia observed in these animals during the experiment. Bodyweight change is used as a general indicator of the adverse effects of chemicals on a living organism [6]. Thus, weight loss is correlated with the physiological condition of the animal and can be explained not only by anorexia [50] but also by impaired metabolism in animals [51]. These data indicate that *R. coccinea* may have adverse effects on animal metabolism. In addition, biochemical parameters related to the liver and kidney functions and hematological parameters are generally explored in the study of the toxicity of medicinal plants. It should be noted that ALAT and ASAT are important markers of liver function. Under normal physiological conditions, these markers are present at low concentrations in the serum. The elevation of serum levels of these enzymes, particularly ALAT, is considered a sensitive marker of liver lesions [52, 53]. Similarly, uremia and creatinine are markers of renal function. Any increase in their serum levels reflects a probable tissue damage to the kidneys [54].

Moreover, the hematopoietic system is one of the most sensitive targets for toxic substances [55]. It is an important marker of the physiological and pathological state of humans and animals. Any alteration in erythrocyte parameters is perceived as a potential risk for anemia [51]. In this study, it should be noted that different treatments have some influence on biochemical markers of renal and hepatic functions. Indeed, there was an increase in alanine aminotransferase (ALAT) levels and a significant increase in uremia in animals treated with *Cassytha filiformis*. This significant increase in uremia was also observed in rats treated with *Rourea coccinea* at doses of 400 mg/kg and 800 mg/kg. However, rats treated with *Gardenia ternifolia* showed a nonsignificant decrease in alanine aminotransferase (ALAT) levels and a significant increase in uremia. In addition, an influence on the hematopoietic system marked by a reduction in the hematocrit level, the number of red blood cells, the mean globular volume, the hemoglobin level, and the mean corpuscular concentration of hemoglobin was also noted for the treatment with *R. coccinea*. These data suggest possible alterations of renal and hepatic functions and risk of anemia. However, histological examination of the kidneys and liver did not reveal any structural alterations of these organs. The confrontation of the data in this study with those in the literature shows variable relationships. Indeed, the studies of Adeyemi et al. [56], on the subacute toxicity of the aqueous extract from the leaves of the plant, reported that daily exposure of Wistar rats to this extract by oral route at doses of 40, 200, and 1000 mg/kg for 30 days had no significant influence on the hematological and biochemical parameters of these animals. However, at an exposure time of 60 days, significant changes in these parameters and bodyweight were reported. Moreover, Wazis et al. [57] reported the toxicity of the ethanolic extract of the leaves of the plant administered by the intraperitoneal route to mice (DL_50_ = 282.24 mg/kg) and Wistar rats (DL_50_ = 1 g/kg). In addition, in this study, no mortality was recorded for animals treated with this plant. These observations indicate that the use of *R. coccinea* at high doses in traditional medicine requires special attention.

Concerning *C. filiformis*, a significant increase in uremia and nonsignificant increase in alanine aminotransferase (ALAT) observed in this study suggest a possible impairment of hepatic and renal functions. Similar data were reported in literature. Indeed, Armenia et al. [58] indicated in an acute toxicity study that the ethanolic extract of the plant at doses of 100 and 400 mg/kg induced hepatic and renal functions in Wistar rats. Similar observations were reported by Yuliandra et al. [59] who showed that the aqueous extract of *C. filiformis* at doses of 2.5, 5, and 10 mg/kg exhibited reversible hepatotoxicity indicated by increased alanine aminotransferase (ALAT) enzyme activity. This trend contrasts with data reported by Babayi et al. [36] in Nigeria. These authors, in assessing the subacute toxicity of the watery extract of *C. filiformis* to Wistar rats, indicated that this type of extract from the plant at doses of 250, 500, and 1000 mg/kg of live body weight did not influence the biochemical and hematological parameters of the animals or alter vital organs such as the heart, liver, and kidney. This observed difference could be explained by the influence of biotic and edaphic conditions (species, plant physiological status, chemical composition, and soil factors).

Concerning *G. ternifolia*, the results of this study indicated that no alteration of hematological parameters was observed. However, a possible renal impairment is to be reported in view of the increase in uremia. In the literature, no studies were specifically carried out on repeated dose toxicity of *G. ternifolia* roots. This study therefore bridged the gap in data on the toxicological profile of the plant. Moreover, two scientific studies of the subacute toxicity of fruit and the acute toxicity of the bark of the roots of *G. ternifolia* were identified in the literature. Indeed, the studies of Farah et al. [60] showed plant fruit safety at 50 mg/kg but altered hematological and biochemical parameters at 500 mg/kg. Nureye et al. [17] showed that the bark of *G. ternifolia* roots was safe for mice at a single dose of 2000 mg/kg. Moreover, the alterations of the various biochemical and hematological parameters observed in this study may be related to the alkaloids present in the parts of plants studied [28, 61, 62]. Indeed, some plant alkaloids such as pyrrolizidine, tropane, piperidine, and indolizidine are known to have toxicological effects [63].

In view of the above mentioned data, it is necessary to recommend the moderate use of this plant, although no mortality was reported in this study. More in-depth toxicological studies (chronic and subchronic toxicity) are necessary for a better understanding of the toxicological profile of *R. coccinea, G. ternifolia*, and *C. filiformis*.

## 5. Conclusion

The purpose of this study was to generate recent data on the toxicological profile of *R. coccinea* leaves, *G. ternifolia* roots, and *C. filiformis* whole plant. The results obtained following the larval cytotoxicity model indicated that the studied plants were not cytotoxic. Data from subacute toxicity indicate that the studied plants did not induce any mortality or structural alterations in the liver and kidneys. However, disorders of certain biochemical and hematological parameters were noted in animals treated with *R. coccinea* and *C. filiformis* at high doses (400 mg/kg and 800 mg/kg). These data suggest a moderate use of these medicinal plants in traditional medicine. Furthermore, toxicological studies (chronic and subchronic toxicity) are needed to better understand the toxicological profile of these plants.

## Figures and Tables

**Table 1:** Effect of studied plants extracts on hematological parameters of rats in different lots.

	Control	*C. filiformis,* 200 mg/kg	*C. filiformis,* 400 mg/kg	*C. filiformis,* 800 mg/kg	*R. coccinea,* 200 mg/kg	*R. coccinea,* 400 mg/kg	*R. coccinea,* 800 mg/kg	*G. ternifolia,* 200 mg/kg	*G. ternifolia,* 400 mg/kg	*G. ternifolia*, 800 mg/kg
RB (T/L)	7.33 ± 0.88	7.01 ± 1.27	7.71 ± 0.42	7.70 ± 0.55	7.44 ± 1.02	5.92 ± 2.40	5.48 ± 1.77	6.73 ± 1.03	6.67 ± 0.42	6.52 ± 1.68
Hb (g/dL)	14.44 ± 1.70	12.55 ± 1.98	14.3 ± 0.45	13.95 ± 0.65	13.67 ± 2.1	11.93 ± 3.92	10.57 ± 3.35	13.56 ± 0.19	15.07 ± 0.65	12.40 ± 1.27
Hte (%)	53 ± 1.73	43.25^*∗*^ ± 2.62	47.25^*∗*^ ± 1.25	47.5^*∗*^ ± 3.11	41.97^*∗*^ ± 4.20	38.23^*∗*^ ± 6.21	33.23^*∗*^ ± 11.33	50.25 ± 2.21	48.5 ± 3.69	47.75 ± 7.80
MGV (fL)	76.33 ± 15.69	62.75 ± 10.37	61.25 ± 4.03	62.25 ± 4.64	56.3^*∗*^ ± 5.02	70.83 ± 23.46	60.53 ± 4.55	78.25 ± 13.09	89.25 ± 2.5	74.75 ± 8.26
MCH (g/dL)	20 ± 5.29	18.25 ± 0.95	18.75 ± 0.5	18.5 ± 0.58	33.1 ± 2.08	30.6 ± 5.91	32.03 ± 1.25	20.75 ± 3.20	26.75 ± 2.36	20 ± 4.69
MCHC (pg)	27 ± 2.64	29.5 ± 3.69	30.5 ± 1.29	29.75 ± 1.25	18.23 ± 1.75	20.77 ± 2.30	19.37 ± 0.93	27 ± 0.81	31.25 ± 2.87	26.5 ± 3.87
WB (T/L)	9.97 ± 1.45	6.925 ± 1.54	9.45 ± 2.7	9.825 ± 1.75	8 ± 2.36	8.37 ± 2.28	10.6 ± 2.65	8.225 ± 4.22	8.4 ± 2.04	8.47 ± 3.89
PN (%)	26 ± 25.53	12.25 ± 10.71	19.25 ± 10.90	9.5 ± 3.87	14.63 ± 3.45	14.1 ± 2.42	11.53 ± 1.23	24.75 ± 14.38	24 ± 3.91	25.25 ± 16.96
Monocytes (%)	5.5 ± 4.95	2.75 ± 2.06	5.25 ± 2.63	3.75 ± 1.5	8.9 ± 7.00	3.63 ± 3.04	2.3 ± 3.16	5.5 ± 2.08	6.25 ± 1.71	5 ± 0.82
Lymphocyte (%)	69.67 ± 27.02	83.5 ± 14.47	72.5 ± 12.92	83.75 ± 5.67	76.47 ± 9.59	82.27 ± 4.72	86.17 ± 4.30	66.5 ± 15.80	47.75 ± 7.13	69.5 ± 17.82

RB, red blood cells; Hb, hemoglobin; Hte, hematocrit; MGV, mean globular volume; MCH, mean corpuscular hemoglobin; MCHC, mean corpuscular hemoglobin concentration; WB, white blood cell; PN, neutrophilic polynuclear. ^∗^Statistically significant.

**Table 2:** Effect of studied plants extracts on biochemical parameters of rats in different lots.

		Uremia (g/L)	Creatinemia (mg/L)	ASAT (UI/L)	ALAT (UI/L)
Control	Day 0	0.34 ± 0.03	7.47 ± 0.46	147.5 ± 19.36	144.5 ± 68.22
	Day 28	0.36 ± 0.04	7.77 ± 0.47	154.5 ± 17.92	106.5 ± 24.79

*C. filiformis*, 200 mg/kg	Day 0	0.37 ± 0.02	6.49 ± 0.53	124.75 ± 19.62	158.25 ± 53.24
	Day 28	0.43 ± 0.05	6.62 ± 0.56	65.25 ± 57.24^*∗*^	175.25 ± 32.16

*C. filiformis*, 400 mg/kg	Day 0	0.39 ± 0.05	7.40 ± 1.06	141.25 ± 23.93	146.12 ± 70.45
	Day 28	0.51 ± 0.03^*∗*^	7.91 ± 1.52	42.5 ± 23.06^*∗*^	155 ± 40.50

*C. filiformis*, 800 mg/kg	Day 0	0.37 ± 0.03	6.83 ± 1.58	132 ± 9.09	104 ± 23.17
	Day 28	0.55 ± 0.10^*∗*^	7.125 ± 1.84	23.25 ± 16.46^*∗*^	110.75 ± 29.85

*R. coccinea,* 200 mg/kg	Day 0	0.39 ± 0.01	6.42 ± 0.29	134.67 ± 11.37	123 ± 14.11
	Day 28	0.44 ± 0.06	6.61 ± 0.32	15.67 ± 4.51^*∗*^	144.67 ± 41.02

*R. coccinea*, 400 mg/kg	Day 0	0.38 ± 0.03	5.39 ± 1.49	146.67 ± 17.56	142.67 ± 83.93
	Day 28	0.58 ± 0.05^*∗*^	5.47 ± 1.51	53.33 ± 40.50^*∗*^	177.67 ± 64.06

*R. coccinea*, 800 mg/kg	Day 0	0.35 ± 0.03	5.29 ± 1.21	136 ± 5.29	190.33 ± 42.03
	Day 28	0.59 ± 0.06^*∗*^	5.51 ± 1.39	91.33 ± 23.85^*∗*^	196.33 ± 26.31

*G. ternifolia*, 200 mg/kg	Day 0	0.36 ± 0.04	7.46 ± 0.48	231.75 ± 50.43	126 ± 65.79
	Day 28	0.43 ± 0.03	8.05 ± 0.91	235.75 ± 52.80	132 ± 97.01

*G. ternifolia*, 400 mg/kg	Day 0	0.39 ± 0.01	7.52 ± 0.48	221.5 ± 44.22	173.25 ± 93.18
	Day 28	0.49 ± 0.03^*∗*^	7.45 ± 0.43	246 ± 68.14	131.75 ± 120.35

*G. ternifolia*, 800 mg/kg	Day 0	0.37 ± 0.03	7.8 ± 0.79	170 ± 31.62	78.25 ± 12.34
	Day 28	0.60 ± 0.10^*∗*^	8.32 ± 1.32	170.5 ± 40.83	71 ± 12.67

^*∗*^ Significantly different.

## Data Availability

The data used to support the findings of this study are included within the article.

## References

[1] OMS (2002). *Stratégie de l’OMS Pour la Médecine Traditionnelle Pour 2002–2005*.

[2] Bayaga H. N., Guedje N. M., Biye E. H. (2017). Approche ethnobotanique et ethnopharmacologique des plantes utilisées dans le traitement traditionnel de l’ulcère de buruli à Akonolinga (Cameroun). *International Journal of Biological and Chemical Sciences*.

[3] Klotoé J., Dougnon T. V., Ategbo J.-M. (2013). Ethnopharmacological survey on antihemorrhagic medicinal plants in south of Benin. *European Journal of Medicinal Plants*.

[4] Béné K., Camara D., Fofie N. (2016). Etude ethnobotanique des plantes médicales utilisées dans le département de Transua, District du Zanzan. *Journal of Animal & Plant Sciences*.

[5] Zerbo P., Millogo-Rasolodimby J., Nacoulma-Ouedraogo O. G. (2007). Contribution à la connaissance des plantes médicinales utilisées dans les soins infantiles en pays San, au Burkina Faso. *International Journal of Biological Chemical Sciences*.

[6] Hilaly J. E., Israili Z. H., Lyoussi B. (2004). Acute and chronic toxicological studies of Ajuga iva in experimental animals. *Journal of Ethnopharmacology*.

[7] Zeggwagh A. A., Lahlou Y., Bousliman Y. (2013). Enquete sur les aspects toxicologiques de la phytotherapie utilisee par un herboriste à Fes, Maroc. *The Pan African Medical Journal*.

[8] Akoègninou A., Van Der Burg W. J., Van Der Maesen L. J. G. (2006). *Flore Analytique du Bénin*.

[9] Agbodjento E., Klotoé J. R., Sacramento T. I. (2020). Ethnobotanical knowledge of medicinal plants used in the treatment of male infertility in southern Benin. *Advances in Traditional Medicine*.

[10] Klotoé J. R., Agbodjento E., Dougnon V. T. (2020). Exploration of the chemical potential and antioxidant activity of some plants used in the treatment of male infertility in southern Benin. *Journal of Pharmaceutical Research International*.

[11] Karou S. D., Tchacondo T., Djikpo Tchibozo M. A. (2011). Ethnobotanical study of medicinal plants used in the management of diabetes mellitus and hypertension in the central region of Togo. *Pharmaceutical Biology*.

[12] Tekou E., Mouzou A. P., Titrikou S., Eklu-Gadegbeku K., Aklikokou K., Gbeassor M. (2012). Effets de l’extrait semi-ethanolique des feuilles de *Gardenia ternifolia* (Rubiaceae), shum et thunn sur le systeme cardiovasculaire du rat wistar. *Journal de la Recherche Scientifique de l’Université de Lomé*.

[13] Holaly G. E., Simplice K. D., Charlemagne G. (2015). Étude ethnobotanique des plantes utilisées dans le traitement du diabète dans la médecine traditionnelle de la région Maritime du Togo. *The Pan African Medical Journal*.

[14] Mpiana P. T., Ngbolua K. T. N., Tshibangu D., Tsalu P. V., Mwanangombo D. T. (2015). Antisickling and radical scavenging activities of anthocyanin extracts from the leaves of *Gardenia ternifolia* subsp. Jovis-Tonantis (Welw.) Verdc. (Rubiaceae). *Sickle Cell Disease Genetics, Management and Prognosis*.

[15] Tshibangu D., Divakar S., Ramanathan M. (2016). In vitro screening of the leaf extracts from *Gardenia ternifolia* (Forest Gardenia) for their anticancer activity. *Journal of Complementary and Alternative Medical Research*.

[16] Ngbolua K. N., Mandjo B. L., Munsebi J. M. (2016). Etudes ethnobotanique et écologique des plantes utilisées en médecine traditionnelle dans le District de la Lukunga à Kinshasa (RD Congo). *International Journal of Innovation and Scientific Research*.

[17] Nureye D., Engidawork E., Assefa S., Nedi T. (2018). In vivo antimalarial activity of the 80% methanolic root bark extract and solvent fractions of *Gardenia ternifolia* Schumach. & Thonn. (Rubiaceae) against *Plasmodium berghei*. *Evidence-based Complementary and Alternative Medicine*.

[18] Farah H. M., Hassan S. M., El Amin T. H., Abdel R. M. E. H. (2016). In vitro activity of the aqueous extract of *Gardenia ternifolia* fruits against *Theileria lestoquardi*. *Journal of Medicinal Plant Research*.

[19] Magassouba F. B., Diallo A., Kouyaté M. (2007). Ethnobotanical survey and antibacterial activity of some plants used in Guinean traditional medicine. *Journal of Ethnopharmacology*.

[20] Oulare K., Barry M. S., Bah F. (2014). Anti-oxidative activity of fruit extracts of some medicinal plants used against chronic diseases (diabetes, hypertension) in Kankan, Guinea. *Journal of Plant Sciences*.

[21] Dahiru Y. Y. D. (2015). Effect of aqueous leaves extract of *Gardenia ternifolia* plant on carbon tetrachloride-induced hepatotoxicity in rats. *IOSR Journal of Pharmacy and Biological Sciences*.

[22] Thiam M. M. (2002). Contribution à l’étude de la valorisation et de la conservation “ex situ” de deux plantes de la pharmacopée traditionnelle sénégalaise: *Combretum micrantum* et *Gardenia ternifolia*.

[23] Diouf A. (2005). Contribution à l’étude chimique d’une plante utilisée au sénégal contre le paludisme “*Gardenia ternifolia*”.

[24] Ochieng C. O., Ogweno Mid J., Okinda Owu P. (2010). Anti-plasmodial and larvicidal effects of surface exudates of *Gardenia ternifolia* aerial parts. *Research Journal of Pharmacology*.

[25] Dada O. K., Akindele A. J., Morakinyo O. A., Sofidiya M. O., Ota D. (2013). Hypoglycemic and antioxidant activities of the hydroethanolic leaf extract of *Byrsocarpus coccineus* Schumach. & Thonn. (Connaraceae). *Chinese Journal of Natural Medicines*.

[26] Akindele A. J., Adeyemi O.O. (2007). Antipyretic activity of *Byrsocarpus coccineus* Schum and Thonn. (Connaraceae). *International Journal of Pharmacology*.

[27] Akindele A. J., Adeyemi O. O. (2006). Evaluation of the antidiarrhoeal activity of *Byrsocarpus coccineus*. *Journal of Ethnopharmacology*.

[28] Ahmadu A. A., Akpulu I. N., Hassan H. S., Sule M. I, Pateh U. U. (2006). Preliminary phytochemical and antimicrobial screening of the leaves of *Byrsocarpus coccineus* Schum & Thonn. (Connaraceae). *Journal of Pharmacy Bioressource*.

[29] Bero J., Ganfon H., Jonville M.-C. (2009). In vitro antiplasmodial activity of plants used in Benin in traditional medicine to treat malaria. *Journal of Ethnopharmacology*.

[30] Adjanohoun E. J., Adjakidjè V., Ahyi M. R. (1986). *Contribution aux Études Ethnobotanique et Floristique en Republique Populaire du Togo*.

[31] Dosseh K., Kpatcha T., Adjrah Y. (2014). Anti-inflammatory effect of *Byrsocarpus coccineus* Schum. and Thonn. (Connaraceae) root. *World Journal of Pharmaceutical Research*.

[32] Lewis W. H. (1986). The useful plants of west tropical Africa. *Economic Botany*.

[33] Hamid A., Aiyelaagbe O. O. (2011). The evaluation of antimicrobial properties and phytoconstituent screening of *Brysocarpus coccineus* leaves grown in south-west Nigeria. *Pelagia Research Library*.

[34] Muhammad B., Mann A., Ndamitso M. M. (2017). GC-MS analysis of phytoconstituents and aphrodisiac activity of the root extract of *Byrsocarpus coccineus* Schum and Thonn. *Lapai Journal of Applied and Natural Sciences*.

[35] Mythili Y., Gajalakshmi S., Sathiavelu A. (2011). Pharmacological activities of *Cassytha filiformis*: a review. *Asian Journal of Plant Science & Research*.

[36] Babayi H. M., Udeme J. J. I., Abalaka J. A. (2007). Effect of oral administration of aqueous whole extract of *Cassytha filiformis* on haematograms and plasma biochemical parameters in rats. *Journal of Medical Toxicology*.

[37] Quetin-Leclercq J., Stevigny C., Hoet S., Block S., Wautier M.C. Studies on Cassytha filiformis from Benin: isolation, biological activities and quantification of aporphines.

[38] Sathiavelu M., Arunachalam S. (2012). High performance thin layer chromatography profile of *Cassytha filiformis*. *Asian Pacific Journal of Tropical Biomedicine*.

[39] Edewor T. I., Owa S. O., Ologan S. O., Ologan A. O., Akinfemi F. (2016). Quantitative determination of the saponin content and GC-MS study of the medicinal plant *Cassytha filiformis* (Linn.) leaves. *Journal of Coastal Life Medicine*.

[40] Swargiary A., Daimari M., Sarma P. (2008). Acute and subacute toxicity evaluation of methanolic extract of *Hodgsonia heteroclita* (Roxb.). *International Journal of Biosciences*.

[41] OCDE_407 (2008). *Test No. 407: Repeated Dose 28-Day Oral Toxicity Study in Rodents*.

[42] OCDE_423 (2002). *Test No. 423: Acute Oral Toxicity—Acute Toxic Class Method*.

[43] Dougnon T. V., Bankolé H., Edorh P. A. (2013). Cytotoxicity of leaves and fruits of *Solanum macrocarpon* Linn (Solanaceae) against shrimp larvae (*Artemia salina* Leach). *Research Journal of Recent Sciences*.

[44] Mousseux M. (1995). Test de toxicité sur les larves d’*Artemia salina* et d’entretien d’un élevage de balanes.

[45] Chijioke E. S., Oko A., Osonwa U., Ofobuike E. G. (2019). In vivo antimalarial screening of ethanolic extract of *Cassytha filiformis* and its ameliorative effect on haematological and biochemical parameters altered in *Plasmodium berghei* infected mice. *Journal of Biology,Chemistry and Pharmacy*.

[46] Ezeigbo I. I., Asuzu I. U. (2010). Preliminary evaluation of the antihyperglycaemic properties of medicinal plants in Nsukka Area, Enugu State, Nigeria. *International Journal of Current Research*.

[47] Sodipo O., Abdulrahman F. I., Alemika T. E., Gulani I. A. (2012). Chemical composition and biological properties of the petroleum ether extract of *Solanum macrocarpum* L. (local name: Gorongo). *British Journal of Pharmaceutical Research*.

[48] Lagarto Parra A., Silva Yhebra R., Guerra Sardiñas I., Iglesias Buelaa L. (2001). Comparative study of the assay of *Artemia salina* L. and the estimate of the medium lethal dose (LD50 value) in mice, to determine oral acute toxicity of plant extracts. *Phytomedicine*.

[49] Sánchez-Fortún S., Sanz F., Santa-María A. (1997). Acute sensitivity of three age classes of *Artemia salina* Larvae to seven chlorinated solvents. *Bulletin of Environmental Contamination and Toxicology*.

[50] Betti A. H., Stein A. C., Dallegrave E. (2012). Acute and repeated-doses (28 days) toxicity study of Hypericum polyanthemum Klotzsch ex Reichardt (Guttiferare) in mice. *Food and Chemical Toxicology*.

[51] Mukinda J. T., Syce J. A. (2007). Acute and chronic toxicity of the aqueous extract of *Artemisia afra* in rodents. *Journal of Ethnopharmacology*.

[52] Amacher D. E. (2002). A toxicologist’s guide to biomarkers of hepatic response. *Human & Experimental Toxicology*.

[53] Ramaiah S. K. (2007). A toxicologist guide to the diagnostic interpretation of hepatic biochemical parameters. *Food and Chemical Toxicology*.

[54] Yuliandra Y., Armenia A., Salasa A. N., Ismed F. (2015). Uji toksisitas subkronis ekstrak etanol tali putri (*Cassytha filiformis* L.) terhadap fungsi ginjal tikus. *Jurnal Sains Farmasi & Klinis*.

[55] Manda P., Manda O., Vangah Manda M. O. (2017). Etude des toxicités aigue et subaiguë du remède nature utilise dans le traitement du paludisme. *Revue Ivoirienne des Sciences et Technologie*.

[56] Adeyemi O. O., Akindele A., Nwumeh K. I. (2010). Acute and subchronic toxicological assessment of *Byrsocarpus coccineus* Schum. and Thonn. (Connaraceae) aqueous leaf extract. *International Journal of Applied Research in Natural Products*.

[57] Wazis C. H., Anuka J. A., Timothy S. Y., Zezi A. U., Mohammed G. T., Hussaini I. M. (2012). Acute toxicity and in-vivo effects of leaf extracts of *Byrsocarpus coccineus* Shum & Thonn in pregnant rat uterus. *Journal of Applied Pharmaceutical Science*.

[58] Armenia N., Gustinanda D., Nur Salasa G., Yuliandra Y. (2015). Acute and delayed toxicity study of *Cassytha filiformis* defatted ethanolic extract. *World Journal of Pharmacy and Pharmaceutical Sciences*.

[59] Yuliandra Y., Armenia A., Arief R., Jannah M. H., Arifin H. (2019). Reversible hepatotoxicity of *Cassytha filiformis* extract: experimental study on liver function and propofol-induced sleep in mice. *Pharmacognosy Journal*.

[60] Farah H., Khalid H., Hussein A., Osman H. (2018). Toxic effect of *Gardenia ternifolia* fruit on rats. *European Journal of Medicinal Plants*.

[61] Ngbolua K. N., Tshibangu D. S. T., Mpiana P. T. (2014). Anti-sickling and antibacterial activities of some extracts from *Gardenia ternifolia* subsp. Jovis-tonantis (Welw.) Verdc. (Rubiaceae) and *Uapaca heudelotii* Baill. (Phyllanthaceae). *Journal of Advances in Medical and Pharmaceutical Sciences*.

[62] Chang F.-R., Chao Y. C., Teng C.-M., Wu Y.-C. (1998). Chemical constituents from *Cassytha filiformis* II. *Journal of Natural Products*.

[63] Adibah K. Z. M., Azzreena M. A. (2019). Plant toxins: alkaloids and their toxicities. *GSC Biological and Pharmaceutical Sciences*.

